# Evaluation of Ghost Cell Survival in the Area of Radiofrequency Ablation

**DOI:** 10.1371/journal.pone.0053158

**Published:** 2012-12-31

**Authors:** Qi Wang, Jiansheng Huang, Kuansheng Ma, Tingjun Li, Ming Chen, Shugang Wang, Ping Bie, Zhenping He

**Affiliations:** The Institute of Hepatobiliary Surgery, Southwest Hospital, Third Military Medical University, Chongqing, P. R. China; University of Birmingham, United Kingdom

## Abstract

**Background and Aim:**

Researchers have demonstrated dead cells in radiofrequency ablation (RFA) lesions that have morphological similarities to viable tumor cells and are thus referred to as ghost cells. However, studies on how long ghost cells persist have not been systematically performed.

**Methods:**

A tumor model was established by implanting VX2 tumor tissue into the livers of 48 New Zealand rabbits. Two weeks later, these tumors were eliminated with RFA. The lesions were resected at 0 weeks, 1 week, 2 weeks, 4 weeks, 8 weeks, or 12 weeks after treatment, and samples were stained either with hematoxylin and eosin (HE) or nicotinamide adenine dinucleotide (NADH). The presence of the cells and the morphological changes that they underwent were examined by light microscopy.

**Results:**

Four weeks after RFA, there were no obvious morphological changes observed in HE-stained ghost cells, and NADH staining revealed no viable cells. Eight weeks after RFA, the cell structure became indistinct. Twelve weeks after RFA, ghost cells were no longer present.

**Conclusions:**

The morphological characteristics of ghost cells are maintained for at least 4 weeks, during which time HE staining cannot be used to differentiate ghost cells from residual tumor cells. NADH staining for cell viability is necessary to differentiate residual tumor cells from ghost cells. This evidence adds to our understanding of the mechanisms of RFA when used on solid tumors.

## Introduction

Radiofrequency ablation (RFA) is a minimally invasive method currently used to treat tumors throughout the world [1]–[3]. Researchers have found that during the process of hyperthermia, cancer cell enzymes and proteins that are involved in cellular organization are rapidly fixed when the temperature is quickly elevated; the morphological structure of the cells is well retained because they are fixed prior to autolysis. Although dead, treated tissues are morphologically similar to viable tissues. The result is the so-called ghost phenomenon, and these residual cells are called ghost cells [4]–[6].

Because no obvious changes in the morphological characteristics can be observed with hematoxylin and eosin (HE) staining of ghost cells, they are difficult to differentiate from viable tumor cells. It is often not understood why ablation of a tumor is incomplete, especially in patients that have operations for liver resection or liver transplantation after RFA treatment of a liver tumor. If a pathological examination was conducted on the tumor sample after an operation, HE staining would reveal that viable tumor cells existed in the area of ablation, which would result in a misunderstanding. Though the phenomenon of ghost cells is understood, studies evaluating how long ghost cells persist have not been systematically performed. The rabbit liver VX2 tumor model is similar to human liver cancer and is often used in liver cancer research, especially to study changes in the tumor microenvironment after local treatments such as RFA, microwaves, and lasers [7]–[9]. In this work, we use the reduced nicotinamide adenine dinucleotide (NADH) histochemical staining method to correctly differentiate ghost cells from residual VX2 tumor cells, to observe how long ghost cells are present, and to characterize their morphological changes over time.

## Materials and Methods

### Experimental Animals

Forty-eight healthy adult New Zealand rabbits (both sexes, 2.0 kg–2.5 kg, 4 mon–5 mon old) were obtained from the Experimental Rabbit Breeding Center (Chongqing, China). Rabbits were divided into 6 experimental groups of 8 rabbits each, and samples were taken at 0 weeks, 1 week, 2 weeks, 4 weeks, 8 weeks, or 12 weeks after RFA. This experiment was authorized by the Experimental Animal Management Committee of the Third Military Medical University (Chongqing, China).

### Equipment and Reagents

The RF 2000 radiofrequency ablation system (RadioTherapeutics, Mountain View, CA) and an extendable assembled multiple-electrode needle were used to treat the tumors. Pentobarbital sodium for injection, nicotinamide adenine dinucleotide (NAD), phenazine methosulfate (PMS), and nitroblue tetrazolium (NBT) were purchased from Sigma-Aldrich (St. Louis, MO). All other reagents were from Sigma, unless otherwise stated.

### VX2 Tumor Model

VX2 tumors are implantable squamous cell carcinomas derived from a rabbit papilloma induced by the Shope virus [10]–[11]. The tumors used in this experiment were provided by the Experimental Animal Research Center of Chongqing Medical University. Tissue resected from the periphery of the tumor under sterile conditions was cut into approximately 1 mm^3^-sized pieces and placed in sterile normal saline. Pentobarbital sodium (3%) was injected in the auricular vein for anesthesia (1 ml/kg) [12]. The upper abdominal region of the rabbit was shaved, sterilized and draped. A midline incision was made into the abdominal cavity, thereby exposing the liver. At 1 cm to 1.5 cm from the lower edge of the left lobe, ophthalmological tweezers were obliquely inserted into the liver, beneath the capsule, to a depth of 1 cm to create a tunnel with a diameter of 2 mm. The pieces of tumor tissue were implanted into the bottom of this tunnel. The incision was then sutured.

### RFA of Experimental Animals

Two weeks after implantation, when the tumors had reached a diameter of approximately 1.5 cm, the rabbits were treated with RFA. The rabbits were anesthetized with 3% pentobarbital sodium (1 ml/kg) injected into the auricular vein and the abdominal wall was opened with a midline incision to determine whether there were tumors and to measure the tumor size if they were present. The radiofrequency electrode was inserted into the center of the tumor, and the thermal detector electrode was placed at the periphery of the tumor. Radiofrequency treatment was initiated beginning at the lowest power of 20 W, followed by increases of 10 W per minute until maximum impedance was reached and one treatment course was completed. According to Ng KK and colleagues, the temperature around the tumor during the experiment, as detected by a thermal electrode, should exceed 60°C to ensure that the tumor cells were ablated and completely inactivated [13].

The rabbits were killed by injecting air into their auricular veins at 0 weeks, 1 week, 2 weeks, 4 weeks, 8 weeks, or 12 weeks after RFA. The tumors were radially resected from the center of the tumor to the edge, including normal hepatic tissue. Each specimen was divided into two pieces. One was fixed with 4% formaldehyde and sectioned using standard techniques for HE staining, whereas the other was embedded in Tissue-Tek OCT (Sakura Finetek, Tokyo, Japan), instantly frozen in liquid nitrogen, and then sliced into cryostat sections using a cryostat refrigerated microtome (2700-Frigocut, Dako Corporation, USA) for NADH staining.

### HE Staining and Cell Counting

Thin slices of tumor tissue for all cases received in our histopathology unit were fixed in 4% formaldehyde solution (pH 7.0) for periods not exceeding 24 h. The tissues were processed routinely for paraffin embedding, and 4 µm-thick sections were cut and placed on glass slides. Tissue samples were stained with hematoxylin and eosin, and the pathologist Wang Bing and Zhu Jing determined the histological type.

A total of 10 sections in ablation region, each section randomly selected five fields at ×400 magnification and photographed counting the number of cells. The cells of sections independently counted by two pathologists previously uninformed of the ghost cells features and the proportion of different cells in the samples.

### NADH Staining

Lactic dehydrogenase (LDH), which is present largely in the cytoplasm of viable cells, catalyzes the oxidation of lactic acid into pyruvic acid while NAD+ is reduced to NADH. A hydrogen ion can then be transferred from NADH to NBT, which is subsequently reduced to a blue crystal that precipitates in the cytoplasm. staining with NADH stain, which has an unambiguous binary staining characteristic of positive staining(blue cytoplasmic staining) indicating viable cells and nonstaining indicating cellular death. This way was used to prove or disprove cell viability.The incubating solution (100 mg of NBT; 80 ml of 0.1 M phosphate buffered saline, pH 7.8–8.0; 10 mg of PMS; 20 ml of 4% sodium lactate; and 50 mg of NAD) was filtered through rough filter paper. Cryostat tissue sections with a thickness of 5 µm were placed on glass slides, washed with double distilled water, and placed in the incubating solution (37°C) for 30 min. The samples were washed with double distilled water and air-dried at room temperature before being covered with glycerogelatin.

## Results

### General Conditions

All 48 New Zealand rabbits in this experiment successfully underwent implantation of VX2 tumors. All tumors were found in the left lobe, and intra-hepatic metastases were found in two rabbits. The VX2 tumor extruded from the hepatic surface and appeared as a grey nodular hard mass with necrosis at the center of some tumors. The cells at the periphery of the tumors were proliferating, the tumor diameters were approximately 1.5 cm, and the tumor was clearly demarcated from the surrounding liver tissue. After RFA, all rabbits demonstrated reduced appetites and manifested inertia and lethargy, but they all recovered after 3 days. During the first 4 weeks, one rabbit died from an intra-abdominal infection.

### Differentiation of Ghost Cells from Viable Cells

**Figure 1:**
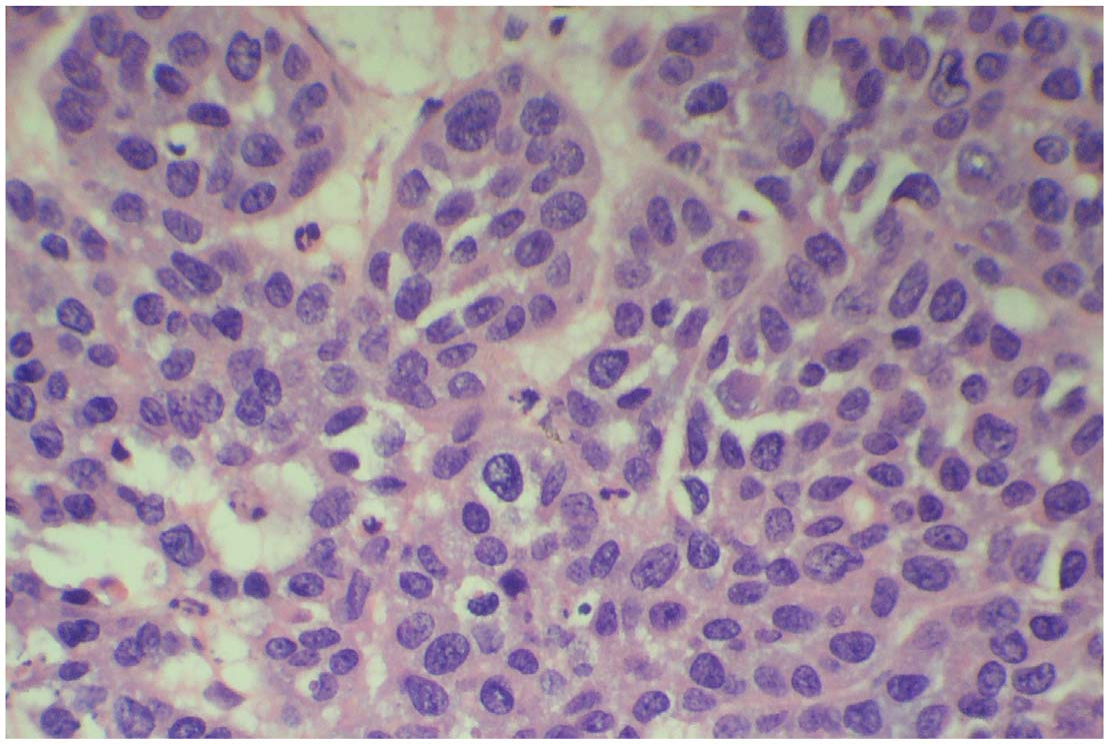
Morphology of VX-2 tumor cells. HE staining of VX-2 tumors implanted in rabbits prior to RFA treatment reveals a poorly differentiated squamous cell carcinoma (magnification×400).

HE staining revealed a clear difference between tumor tissue and parenchymal tissue. In the staining of VX2 tumor cell before the RFA, some tumor cells(viable tumor cells) assembled into clusters; their structure was disorderly, and necrosis-like changes were found in some cells at the center of the tumor, probably due to ischemia. At 400× magnification, tumor cells appeared as a poorly differentiated squamous cell carcinoma with obvious atypia, irregular morphology, increased size, a disordered cell nucleus with varying volume and shape, and pathological nuclear divisions. Increased numbers of unevenly distributed chromosomes and enlarged nucleoli were also found within the deeply stained nucleus (Fig. 1).

**Figure 2:**
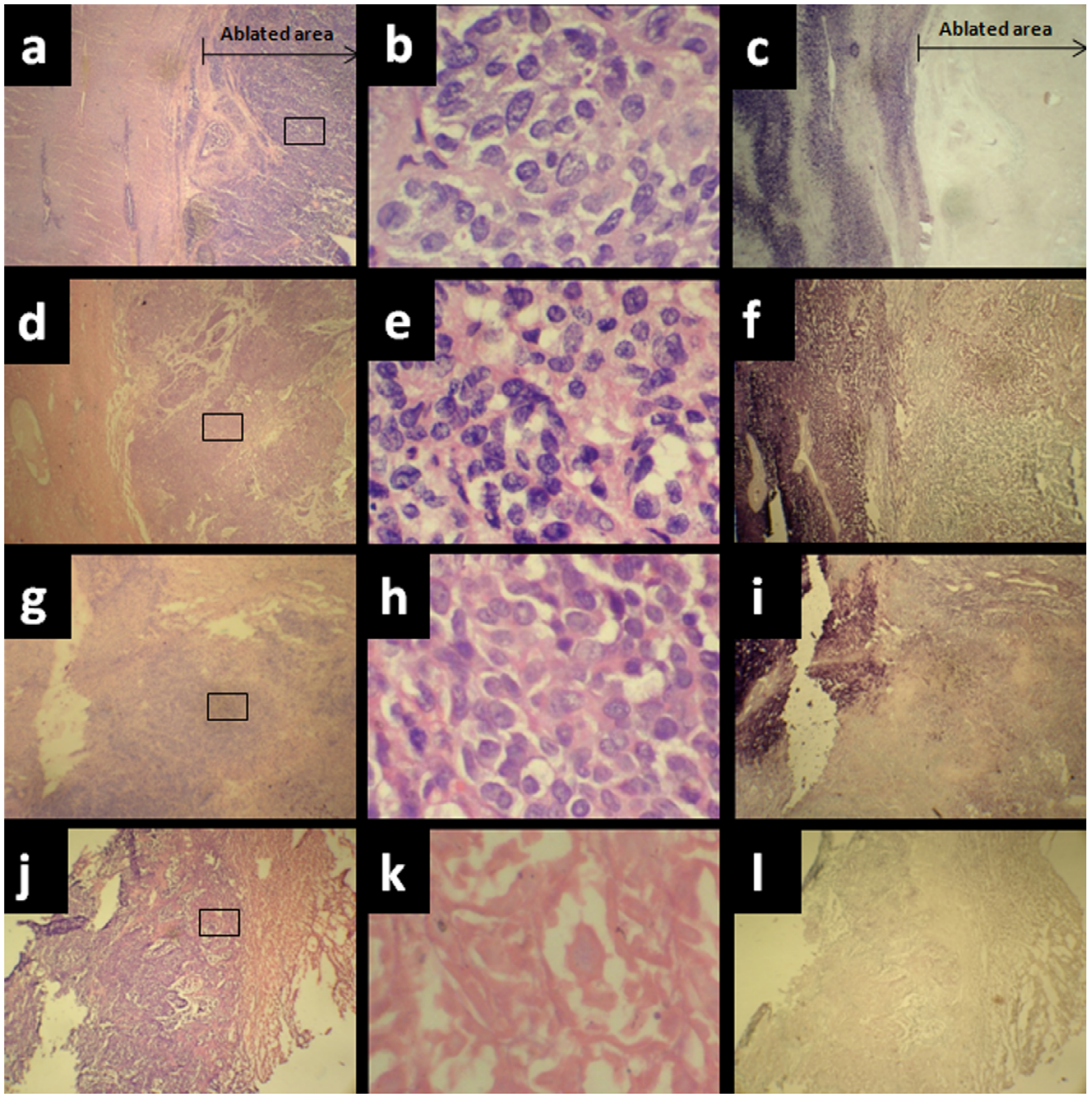
(a–c) VX-2 tumors from rabbits immediately after RFA treatment. After RFA treatment, as described in the Materials and Methods section, rabbits were killed, tumors were excised, and samples were stained with HE and NADH. (a) HE staining, magnification×40; (b) HE staining, magnification×400; (c) NADH staining, magnification× 40.(d–f) VX-2 tumors from rabbit 2 weeks after RFA treatment. (d) HE staining, magnification×40; (e) HE staining, magnification×400; (f) NADH staining, magnification× 40.(g–i) VX-2 tumors from rabbit 4 weeks after RFA treatment. (g) HE staining, magnification×40; (h) HE staining, magnification×400; (i) NADH staining, magnification×40. (j–l) VX-2 tumors from rabbits 12 weeks after RFA treatment. (j) HE staining, magnification×40; (k) HE staining, magnification×400; (l) NADH staining, magnification×40.

Immediately following the RFA, at 40× magnification, the ablation border reached the normal hepatic tissue at the edge of the tumor. A large area of tissue necrosis was observed in the ablation region(already by visual inspection, the necrosis area going gray and hard). Damaged cells with only the outer edge visible, fragmented and disintegrated nuclei, and coagulation of the blood vessels were all visible in the congested periphery of the ablation region (Fig. 2a). When the adjacent resected section was stained with NADH, blue-colored viable tissue was visible only outside the ablation region, indicating that the targeted cells had lost their metabolic activity (Fig. 2c). However, at 400× magnification, some non-viable cells were seen scattered throughout the ablation region (Fig. 2b). These cells were not obviously different from viable tumor cells (Fig. 1). The relative proportions of ghost cells in whole cell of ablated area able to achieve 84.3%, and scattered throughout the ablation region. In addition, damaged cells (nuclear fragmentation, disintegrated, concentrated) accounted for about 8.2% and scattered the outer edge of ablation region. coagulation of the blood vessels and Fibrous tissue together accounted for about 7.5% and scattered throughout the ablation region.

One week after RFA, NADH staining revealed that no viable cells existed in the ablation region. However, HE staining revealed the presence of ghost cells with no obvious morphological differences from viable tumor cells. Two weeks after RFA, at 400× magnification, ghost cells were still seen scattered throughout the ablation region (Fig. 2e), which, as determined by NADH staining, were not viable (Fig. 2f). a massive amount of Collagen was seen outside the damaged region. Four weeks after RFA, NADH staining revealed that the ablation region no longer contained viable cells (Fig. 2i), while HE staining revealed ghost cells that were morphologically similar to viable tumor cells (Fig. 1), except for a lightly stained nucleus and cytoplasm (Fig. 2h). collagen fibers was proliferated and dense, which encircled the ablation region, was obvious in the damaged area. Eight weeks after RFA, only the outline of some residual necrotic tumor cells could be found in the coagulative necrotic lesion. The general structure was difficult to determine but was obviously different from viable tumor cells. Twelve weeks after RFA, a large area of evenly but lightly dyed red tissue was observed in the ablation region, in which neither tumor cells nor ghost cells could be identified using higher magnification (Fig. 2k).

## Discussion

### Timetable of Ghost Cell Maintenance

The typical ablation region is pathologically divided into three concentric circles with the electrode at the center: Region A, the carbonized area around the electrode; Region B, the paler area of the ablation lesion; and Region C, the hyperemia band [14]–[15]. Our results demonstrated that ghost cells were present from the day of RFA in the damaged area between the hyperemia band and the carbonized area around the central electrode track. This type of non-viable cell exists up to 4 weeks post-RFA in smaller numbers but shows no obvious morphological changes, except for lighter cell staining. These ghost cells cannot be definitively differentiated from viable tumor cells by HE staining. The relative proportions of ghost cells in whole cell of ablated area able to achieve 80–90%, and scattered throughout the ablation region. Many factors perhaps affect the proportion of ghost cells. For example, Operative techniques, RFA needle type, The power of the Equipment, etc. However, 8 weeks after RFA treatment, only the outline of some residual necrotic tumor cells, which became less distinct and more lightly dyed, were found in the coagulative necrotic lesion and these necrotic tumor cells could thus be easily differentiated from viable tumor cells. Twelve weeks after RFA, all ghost cells in the ablation region had disappeared.

One possible mechanism to explain the existence of ghost cells is that the coagulative effect of the radiofrequency-induced temperature spike fixes the cell structure and prevents the release of lysosomal enzymes, thereby delaying autolysis. In addition, the cell structure could be preserved because damaged tumor blood vessels make it impossible for inflammatory cells to infiltrate the region and participate in tissue autolysis [16]–[17].

### Significance of NADH Staining

NADH staining is used to evaluate ablation lesions after RFA treatment as a specific and valid method for evaluating the necrotic area of the ablation region [18]–[20]. The cytoplasm of cells in the ablation lesion is not detectable by this technique because the radiofrequency heat damage destroys the LDH. Although some tumors have been radically treated with RFA, some residual tumor is detected upon pathological examination; therefore, ablation treatment cannot be considered radical. HE staining makes it difficult to judge whether the residual tumor tissue is viable. In this experiment, use of the NADH staining method correctly differentiated ghost cells from residual tumor cells.

Technical limitations restricted our analysis of adjacent sections of incised tissue for evaluation by HE or NADH staining. New techniques are needed to observe ghost cells in the same section of incised tissue.
